# Genomic Signature for Initial Brain Metastasis Velocity (iBMV) in Non-Small-Cell Lung Cancer Patients: The Elusive Biomarker to Predict the Development of Brain Metastases?

**DOI:** 10.3390/cancers17060991

**Published:** 2025-03-15

**Authors:** Sarah E. Glynn, Claire M. Lanier, Ariel R. Choi, Ralph D’Agostino, Michael Farris, Mohammed Abdulhaleem, Yuezhu Wang, Margaret Smith, Jimmy Ruiz, Thomas Lycan, William Jeffrey Petty, Christina K. Cramer, Stephen B. Tatter, Adrian W. Laxton, Jaclyn J. White, Jing Su, Christopher T. Whitlow, David R. Soto-Pantoja, Fei Xing, Yuming Jiang, Michael Chan, Corbin A. Helis

**Affiliations:** 1 Department of Radiation Oncology, Wake Forest University School of Medicine, Winston Salem, NC 27157, USA; cmlanier@wakehealth.edu (C.M.L.); archoi@wakehealth.edu (A.R.C.); mfarris@wakehealth.edu (M.F.); ccramer@wakehealth.edu (C.K.C.); yumjiang@wakehealth.edu (Y.J.); mchan@wakehealth.edu (M.C.); chelis@wakehealth.edu (C.A.H.); 2 Department of Biostatistics and Data Science, Wake Forest University School of Medicine, Winston Salem, NC 27157, USA; rdagosti@wakehealth.edu; 3 Department of Medicine, West Virginia University, Morgantown, WV 26506, USA; mohammed.abdulhaleem@hsc.wvu.edu; 4 Department of Molecular and Cellular Bioscience, Wake Forest University School of Medicine, Winston Salem, NC 27157, USA; yuewang@wakehealth.edu (Y.W.); margsmit@wakehealth.edu (M.S.); 5 Department of Internal Medicine, Section of Hematology and Oncology, Wake Forest University School of Medicine, Winston Salem, NC 27157, USA; jruiz@wakehealth.edu (J.R.);; 6 Department of Neurosurgery, Wake Forest University School of Medicine, Winston Salem, NC 27157, USA; statter@wakehealth.edu (S.B.T.); alaxton@wakehealth.edu (A.W.L.); jjwhite@wakehealth.edu (J.J.W.); 7 Department of Biostatistics and Health Data Science, Indiana University School of Medicine, Indianapolis, IN 46202, USA; jsu@wakehealth.edu; 8 Department of Diagnostic Radiology, Wake Forest University School of Medicine, Winston Salem, NC 27157, USA; cwhitlow@wakehealth.edu; 9 Department of Cancer Biology, Wake Forest University School of Medicine, Winston Salem, NC 27157, USA; dsotopan@wakehealth.edu (D.R.S.-P.); fxing@wakehealth.edu (F.X.); 10 Department of Radiation Oncology, Alexander T. Augusta Military Medical Center, Fort Belvoir, VA 22060, USA

**Keywords:** brain metastases, next-generation sequencing, non-small-cell lung cancer, brain surveillance, genetic biomarkers, radiotherapy

## Abstract

**Background/Objectives**: No prior studies have attempted to identify a biomarker for initial brain metastasis velocity (iBMV), with limited studies attempting to correlate genomic data with the development of brain metastases. **Methods**: Patients with non-small-cell lung cancer (NSCLC) who underwent next-generation sequencing (NGS) were identified in our departmental database. iBMV was calculated by dividing the number of BMs by the interval of time between primary cancer and BM diagnosis. Two-sample *t*-testing was used to identify mutations statistically associated with iBMV (*p* < 0.1). A value of +1 was assigned to each mutation with a positive association (“deleterious genes”), and a value of −1 to each with an inverse association (“protective genes”). The sum of these values was calculated to define iBMV risk scores of −1, 0 and 1. Pearson correlation test was used to determine the association between iBMV risk score and calculated iBMV, and a competing risk analysis assessed for death as a competing risk to the development of BMs. **Results**: A total of 312 patients were included in the analysis, 218 of whom (70%) developed brain metastases. “Deleterious genes” included ARID1A, BRAF, CDK4, GNAQ, MLH1, MSH6, PALB2, RAD51D, RB1 and TSC1; “protective genes” included ARAF, IDH1, MYC, and PTPN11. iBMV risk scores of 1, 0 and −1, predicted an 88%, 61% and 65% likelihood of developing a BM (*p* < 0.01). A competing risk analysis found a significant association between iBMV risk scores of 1 vs. 0 and 1 vs. −1, and the likelihood of developing a BM using death as a competing risk. Overall survival (OS) at 1 and 2 years for patients with iBMV risk scores of 1, 0 and −1 was 72% vs. 84% vs. 85% and 46% vs. 69% vs. 70% (*p* < 0.02). **Conclusions**: Development of a genomic signature for iBMV via non-invasive liquid biopsy appears feasible in NSCLC patients. Patients with a positive iBMV risk score were more likely to develop brain metastases. Validation of this signature could lead to a biomarker with the potential to guide treatment recommendations and surveillance schedules.

## Simple Summary

Brain metastases (BMs) are diagnosed in 200,000 people every year, with non-small-cell lung cancer (NSCLC) patients accounting for approximately 50% of cases. These patients are at increased risk of dying from BMs, and timely diagnosis can be challenging due to limited resources and current surveillance guidelines, which recommend no follow-up brain imaging after an initial negative scan, unless patients become symptomatic. Therefore, a potential biomarker to identify patients at high risk for the development of brain metastases could guide monitoring and treatment strategies, improving care for NSCLC patients. We analyzed genetic data from liquid biopsies to identify mutations statistically linked to increased or decreased risk of BM development. Based on these findings, a risk score was created to predict BM risk and survival outcomes. External validation of this novel biomarker could establish a non-invasive, predictive tool to stratify and optimally manage patients with a higher risk of BM development, by placing them into surveillance regimens for the earlier detection of BMs.

## 1. Introduction

Nearly 200,000 patients in the United States are diagnosed with brain metastases (BMs) each year, with lung cancer representing the most common primary site, followed by melanoma, breast and kidney cancers [1,2]. While there has been a migration over the past two decades from large symptomatic BMs to occult BMs discovered from routine staging practices [3], patients who develop symptomatic BMs are still at high risk of dying of BMs, with a median survival time from diagnosis of six months [4].

Patients with non-small-cell lung cancer (NSCLC) represent approximately 50% of patients with BMs [5] and have the highest incidence proportion for developing BMs among all patients with a stage I cancer [6]. In NSCLC patients, a standard approach for surveillance of the brain is to acquire magnetic resonance imaging (MRI) of the brain at diagnosis as part of initial staging for anyone with stage IIB or greater disease, and if that is negative, to only image the brain again if neurologic symptoms arise [7]. This approach may be inadequate in the era of improved systemic therapies, which have prolonged patient overall survival (OS) and consequently increased the time at risk of developing BMs [8]. Some institutions may re-stage the brain at the time of systemic progression of disease; however, given the large number of patients with NSCLC in the US, it is often not feasible or cost-effective to regularly image all NSCLC patients to screen for BMs.

A potential role for a biomarker for BMs is to identify patients at high risk of developing BMs. Several prior attempts have been made to identify such biomarkers [9,10]. Issues that complicate attempts at biomarker identification for the development of BMs include a biologically heterogeneous population, invasive tissue acquisition, and small sample sizes for genetic analysis. Initial brain metastasis velocity (iBMV) is a recently described clinical biomarker that describes the rate at which BMs develop from the time the cancer is originally diagnosed [11]. A potential biomarker to predict iBMV would represent a major advancement in the ability to determine patients at risk of developing BMs. This information could be used to triage these patients to more frequent brain imaging surveillance, leading to earlier BM diagnosis and intervention, to ultimately improve patient neurologic symptoms and complications, overall quality of life, and medical system resource allocation.

Comprehensive genomic profiling using liquid-biopsy acquired analysis of circulating tumor DNA has been shown to be able to predict oncologic outcomes, such as response to chemoimmunotherapy [12] and oligometastatic progression [13]. Smith et al. demonstrated that mutational composite scores were more predictive than tumor mutational burden or PD-L1 status of patient response to immunotherapy [12], while Choi et al. recently showed through external validation that their previously reported genomic profile for oligometastatic disease in patients with brain metastases, predicted oligometastatic disease state (*p* = 0.03) and demonstrated a trend toward the prediction of oligoprogression [13]. Moreover, these analyses have been successful in predicting BM outcomes such as local progression, distant brain progression and lesion size [14]. The present analysis builds on the prior successes of using genomic profiling to assess BM outcomes by seeking a genomic signature for iBMV.

## 2. Materials and Methods

### 2.1. Data Acquisition

This study was conducted at Atrium Health Wake Forest Baptist, Winston-Salem, and was approved by the Wake Forest Health Sciences Institutional Review Board, IRB00033881. A prospective database is kept at our institution for all patients who undergo commercial comprehensive genomic profiling. This database was used to identify all patients diagnosed with NSCLC between 2005 and 2021, who also underwent genomic profiling using the Guardant 360 platform (*n* = 347). Patients who underwent genomic profiling through other platforms were excluded from the study, as were patients with a diagnosis of small-cell lung cancer. Patients without MRI brain (*n* = 30), with equivocal MRI brain findings without confirmation (*n* = 2), with mixed SCLC/NSCLC diagnosis (*n* = 1) and with missing pathology at diagnosis (*n* = 1) were excluded. Of note, institutional standard for acquiring genomic profiling during the timeframe of this study was for a new diagnosis of metastatic or locally advanced NSCLC. In the beginning of the study, when genomic profiling was earlier in its evolution, not every patient underwent genomic profiling; however, it became the standard of care at our institution by 2016. Electronic medical records were used to determine patient demographics at time of diagnosis, including age, sex, race and Karnofsky Performance Status (KPS), as well as clinical outcomes, including the development of BMs. All patient MRIs and clinical notes were reviewed both at staging and throughout their clinical course to determine whether patients developed BMs. Patients who never developed BMs were required to have a minimum follow-up of two years for study inclusion. Data collection ended on 1 February 2024.

### 2.2. Initial Brain Metastasis Velocity

iBMV was calculated based on the previous publication by Soike et al. [11] as detailed below:

iBMV = N/T

where N = the number of BMs at the time of BM diagnosis and T = the time from initial cancer diagnosis to the development of the BM measured in years. For example, if a patient develops two BMs six months after primary diagnosis, iBMV = 2/0.5 = 4. The date of initial cancer diagnosis was defined as the date of pathologic confirmation of malignancy.

An iBMV of 300 was assigned to patients with BMs at time of primary diagnosis and a score of 0 to patients who never developed BMs during a minimum follow-up of two years.

### 2.3. Comprehensive Genomic Profiling

Comprehensive genomic profiling was performed throughout the course of the study period 2005–2021, using a commercially available test from Guardant 360 platform (Guardant Health, Palo Alto, CA, USA). Test samples were acquired via liquid biopsy on peripheral blood. The platform is minimally invasive and assesses circulating tumor DNA for known mutations in NSCLC [15], including AKT1, ALK, APC, AR, ARAF, ARID1A, ATM, BRAF, BRCA1, BRCA2, CCND1, CCNE1, CDH1, CDK4, CDK6, CDK12, CDKN2A, CHEK2, CTNNB1, DDR2, EGFR, ERBB2, ESR1, EZH2, FANCA, FBXW7, FGFR1, FGFR2, FGFR3, GATA3, GNA11, GNAQ, GNAS, HNF1A, HRAS, IDH1, IDH2, JAK2, JAK3, KEAP1, KIT, KRAS, MAP2K1, MAP2K2, MAPK1, MET, MLH1, MPL, MSH2, MSH6, MTOR, MYC, NF1, NFE2L2, NOTCH1, NPM1, NRAS, NTRK1, NTRK2, NTRK3, PALB2, PDGFRA, PIK3CA, PMS2, PTEN, PTPN11, RAD51D, RAF1, RB1, RET, ROS1, SMAD4, SMO, STK11, TERT, TP53, TSC1 and VHL. Blood testing was generally acquired prior to initiation of systemic therapy for metastatic disease, as published by Leigh et al. [16].

### 2.4. Statistical Analysis

The identification of gene mutations of interest was carried out, as previously published by Abdulhaleem et al. [14]. First, a two-sample *t*-testing was used to identify gene mutations associated with iBMV. Since this first step was to screen all genes to identify any that had a potential signal, a higher *p*-value (<0.1) was used to identify potential genes for the signature. Next, each gene that was identified with a *p* < 0.1 was examined to see whether the presence (or absence) of the gene predicted a higher (or lower) iBMV. Based on this evaluation, a value of +1 was assigned to each mutation with a positive association with increased iBMV (“deleterious genes”), and a value of −1 to each mutation present with an inverse association (“protective genes”). The sum of these values was calculated to define iBMV risk scores of −1 (“negative iBMV”), 0 (“neutral iBMV”), with risk scores of 1, 2 and 3 pooled into a single group 1 (“positive iBMV”). A competing risk analysis using a Fine–Gray sub-distribution hazard model was then used to examine the time to brain metastases, while considering death as a competing risk. Cumulative incidence plots were created with corresponding 95% confidence intervals. Prior to fitting this model, we examined the proportional hazards assumption for time to brain metastases by examining Schoenfeld residuals [17]. In addition, a separate Cox proportional hazards model was fit, adjusting for patient’s age, disease stage and smoking status at time of diagnosis, to the impact on the iBMV risk score. Finally, we examined whether the iBMV risk score predicted overall mortality among patients using a Kaplan–Meier survival analysis and compared survival curves using the log rank statistic. Statistical analysis was performed using SAS version 9.4 (SAS Institute, Cary, NC, USA).

## 3. Results

### 3.1. Patient Population

**Figure 1:**
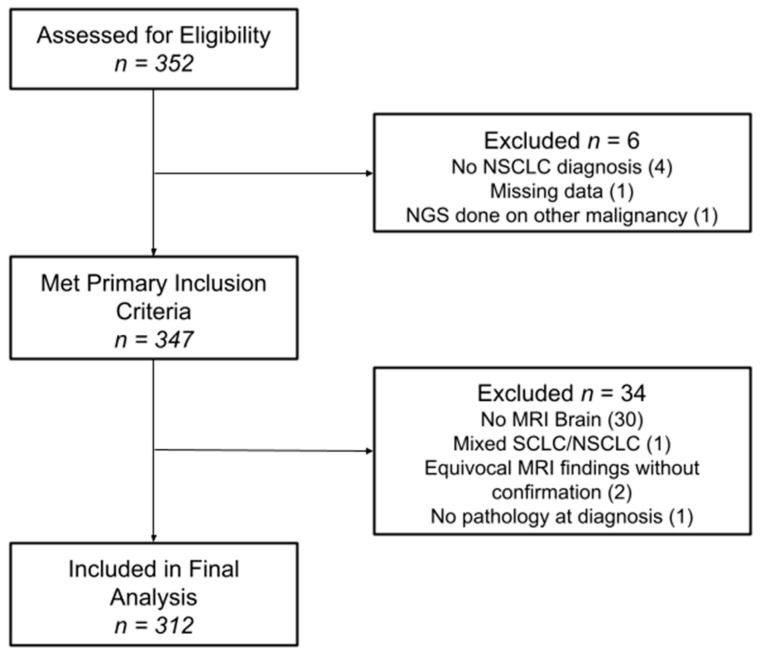
Consort diagram for study inclusion.

Between 2005 and 2021, 312 patients met the study criteria and were included in the final analysis (Figure 1). The median follow-up was 2.6 years, defined as the time from primary cancer diagnosis to the date of the last brain MRI. Patient characteristics at the time of diagnosis are summarized in Table 1 for the entire cohort and by individual iBMV risk score. Patients were evenly distributed between male (49.7%) and female (50.3%), and the median age at diagnosis was 67 years old. The majority of patients were white (84.9%) and African American (12.8%), with KPSs of 80–100% (64.4%) and 50–70% (31.7%). Most patients were former smokers (62.2%), followed by current (22.1%) and never-smokers (15.7%). Adenocarcinoma (79.2%) represented the most common histology, followed by squamous cell carcinoma (17%), NSCLC-NOS (3.2%) and large-cell carcinoma (0.6%). Most patients were diagnosed at advanced stages: stage IV (85.6%), stage III (12.2%), stage II (1.0%) and stage I (1.3%). The most frequent actionable mutation associated with NSCLC was EGFR (22.1%), followed by KRAS (30.1%), BRAF (8.7%), ALK (6.1%), and ROS1 (4.5%). Most patients had a neutral iBMV (63.1%), with positive iBMV (30.4%) the next most frequent and negative iBMV (6.4%) the least common. This relative distribution of iBMV risk scores was consistent both across sex and race. In all, 218 (70%) patients developed BMs.

### 3.2. Development of Genomic Risk Score for iBMV

Genetic mutations in the next-generation sequencing (NGS) panel that were found to be associated with elevated iBMV (“deleterious genes”) included ARID1A, BRAF, CDK4, GNAQ, MLH1, MSH6, PALB2, RAD51D, RB1 and TSC1; those with an inverse association to iBMV (“protective genes”) included ARAF, IDH1, MYC, and PTPN11.

### 3.3. Development of Brain Metastases Based on iBMV Genomic Risk Score

**Figure 2:**
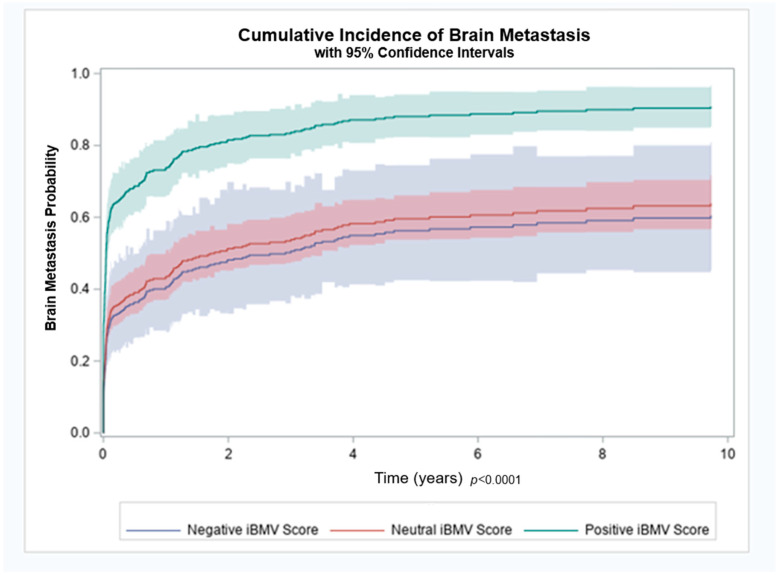
Brain metastasis cumulative incidence for patients with initial brain metastasis velocity (iBMV) risk scores of 1 (positive, green), 0 (neutral, red) and −1 (negative, blue). *p* < 0.0001.

Patients with a positive, neutral and negative iBMV risk score, had an 88%, 61% and 65% likelihood of developing BMs, respectively (*p* < 0.01). Cumulative incidence of brain metastasis at 1 year for patients with a positive, neutral and negative iBMV risk score were 74%, 43% and 40%, respectively, as shown in Figure 2. A competing risk analysis found a statistically significant association between iBMV risk scores of 1 vs. 0 and 1 vs. −1, respectively, and the likelihood of developing BMs using death as a competing risk (HR 2.35, 95% CI 1.77–3.11 and HR 2.57, 95% CI 1.57–4.20, *p* < 0.0001). A proportional hazards regression model that included age, stage and smoking status found that iBMV risk score remained a significant predictor of time to development of BMs (*p* < 0.0001), despite the fact that patient age (*p* < 0.0001) and stage at diagnosis (*p* < 0.0001) were also predictive. Smoking status at time of diagnosis was not a significant predictor of time to development of BMs (*p* = 0.18).

### 3.4. Other Clinical Outcomes Related to iBMV Genomic Risk Score

**Figure 3:**
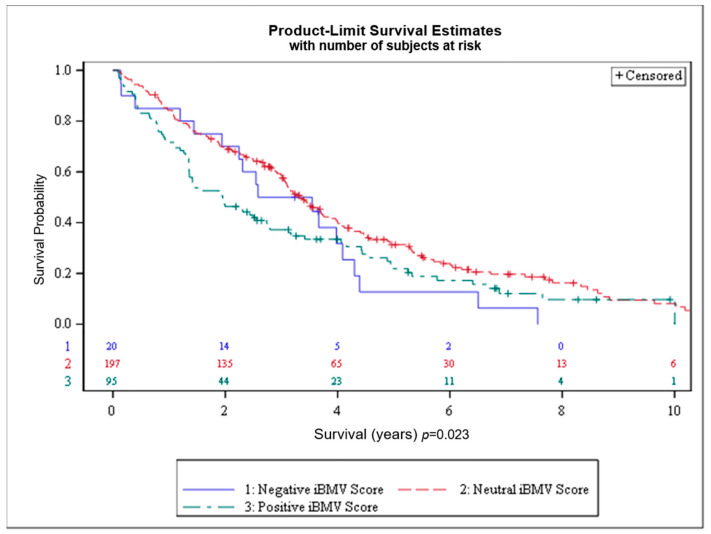
Overall survival (OS) for patients with initial brain metastasis velocity (iBMV) risk scores of 1 (positive, green), 0 (neutral, red) and −1 (negative, blue). *p* < 0.023.

Overall survival by iBMV risk score is shown in Figure 3. OS at 1 and 2 years, respectively, for patients with positive, neutral and negative iBMV risk scores was 72% vs. 84% vs. 85% and 46% vs. 69% vs. 70% (*p* < 0.02). Median survival for patients with positive, neutral and negative iBMV risk scores was 1.95 years (95% CI 1.36–2.74), 3.34 years (95% CI 3.04–3.73) and 3.06 years (95% CI 1.44–4.09).

## 4. Discussion

Within five years of NSCLC diagnosis, approximately 12.6% of patients will develop BMs [18], with multiple studies and tumor registries reporting a BM incidence ranging from 17% to 36% during the disease course [19]. Several publications have suggested that the histology of NSCLC may affect its propensity for seeding the brain [20,21]. A retrospective analysis of 264 patients with NSCLC reported a predicted probability of BMs, when controlling for primary tumor size and nodal status (to correlate positively with cell type) in adenocarcinoma of 0.43 (95% CI: 0.35–0.52) and undifferentiated of 0.36 (95% CI: 0.25–0.48) vs. squamous of 0.10 (95% CI: 0.05–0.22, *p* = 001) [22]. Hsiao et al. determined on multivariate logistic regression that among 482 stage IIIB/IV NSCLC patients, the presence of BMs was associated with adenocarcinoma (OR = 2.39, 95% CI: 1.16–4.92, *p* = 0.018), as well as female gender and age < 60 years old [23].

Prior studies have elucidated several genetic mutations that increase the likelihood of developing brain metastases from NSCLC. These studies have shown the cumulative rate of brain metastases in up to 60% of patients with EGFR mutations, and up to 40% of patients with ALK mutations with a median time to diagnosis of brain metastases of less than one year. This is compared to just greater than 20% for a general cohort of NSCLC patients after two years [24,25,26]. In a retrospective cohort study from South Korea, Shin et al. [27] found that among 314 patients with NSCLC adenocarcinoma who underwent MRI brain at diagnosis, there was a strong association between EGFR mutation status and brain metastasis (adjusted OR = 3.83, *p* = 0.001). ALK translocation is a rarer entity, detected in only 3–7% of NSCLC patients [28]; however, among these patients, synchronous BM diagnosis or CNS progression within months of diagnosis is reportedly as high as 25–40% [1,29]. Despite their high risk for BMs, EGFR- and ALK-mutated cancers collectively represent only 17% of the NSCLC population. Therefore, a genomic signature that affects a greater proportion of patients with brain metastases is clearly warranted. The present data validate that genetic mutations can serve as biomarkers for earlier seeding of the brain and identify several mutations that are related to iBMV.

The clinical potential of a biomarker predictive of iBMV is immense. Current guidelines recommend brain MRI or head CT with contrast at diagnosis for NSCLC patients with stage II and greater disease, with subsequent brain imaging reserved for patients who develop neurologic symptoms and/or progressive disease. A biomarker to identify patients at the time of diagnosis who have a high risk of developing BMs could be used to place this subset of patients into an imaging surveillance program, with the intent of increasing the proportion of patients able to be treated with minimally invasive treatment techniques such as stereotactic radiosurgery (SRS) if BMs develop [30]. The use of SRS for limited BMs has been shown to result in less cognitive decline and better quality of life, as well as a non-significant difference in OS, when compared to SRS plus whole-brain radiotherapy (WBRT) [31]. On the other hand, patients who are at low risk of developing BMs may end up being better managed without imaging surveillance. Recent analyses have demonstrated that the development of BMs significantly increases the cost of management of cancer patients [32]. Therefore, tools that can help triage patients to the most efficient CNS surveillance management paradigm may improve the cost-effectiveness of treatment. Although our group has not yet conducted a clinical feasibility assessment nor cost-effectiveness analysis, these are essential areas of future research to evaluate the clinical utility of our biomarker.

While the original role of genomic profiling was primarily treatment-driven, with a goal of identifying actionable mutations for which targeted drugs exist that can be used instead of traditional cytotoxic chemotherapy, more recently, genomic profiles have been identified to predict clinically meaningful endpoints in oncologic management [13,14]. As previously described, recent advancements in this realm include mutational composite scores to predict response to immunotherapy [12], unique genomic profiles to predict oligometastatic disease [13] and leptomeningeal disease progression [33], as well as multiple genetic signatures associated with other clinical outcomes including brain metastasis velocity (BMV), time to distant brain failure, lowest radiosurgery dose, extent of extracranial metastatic disease, synchronous diagnosis of brain metastasis and NSCLC, number of brain metastases at diagnosis, and distant brain failure [14]. This growing field of research has important implications, as more genomic profiles are validated against external databases, to provide specific prognostic information and to help guide surveillance and therapeutic interventions.

The potential implications of the findings in this series go beyond the clinical utility of having a tool to predict iBMV. Previous studies have suggested that certain genes may increase the likelihood of cancers seeding the brain, irrespective of histology [34]. The genes identified in this series may have potential in reverse translational preclinical studies to elucidate mechanisms for how cancer colonizes the brain. Furthermore, genes that lead to iBMV may be assessed for the potential to predict future BMV [35] or the need for WBRT [36].

Future directions would be to obtain independent and prospective validation of the iBMV signature. As it took several years to amass a population at our institution sufficiently large to generate this dataset, a multi-institutional validation project would be most optimal. Furthermore, it would be beneficial to assess the signature’s validity over multiple genomic platforms, and in both primary tumor tissue and circulating tumor DNA. This could potentially allow for previously obtained tissue to be used for assessment instead of obtaining new tissue at the time of metastatic presentation. Our group additionally plans to conduct clinical feasibility assessments and cost-effectiveness analyses to address these essential aspects of clinical utility. Finally, machine learning algorithms could be used to devise multi-parametric biomarkers for brain metastases, integrating genomic and demographic data, radiomics and pathomics.

There are several limitations to the present series. As a retrospective dataset, it is prone to the patient selection bias of a large academic medical center and needs to be validated by an independent dataset prior to being introduced clinically. For example, the homogeneous racial makeup of the study population (84.9% white) could limit the generalizability of the study results. In addition, it is possible that the signature genes found in the present study’s limited dataset might be rarer in a larger dataset, affecting their predictive value. Over the study period, systemic therapy for metastatic NSCLC changed considerably, such as with the widespread adoption of immune checkpoint inhibitors, which have been associated with a >60% relative increase in median OS among NSCLC brain metastasis patients [37]. These therapeutics likely have intracranial activity and may have impacted the likelihood of patients developing brain metastases.

## 5. Conclusions

iBMV is a recently developed biomarker which describes the rate at which the brain is seeded by metastases. Non-invasive liquid biopsy is a feasible method for developing a genomic signature that predicts iBMV for NSCLC patients. Patients with a high iBMV risk score are more likely to develop brain metastases and have worse overall survival. If validated in future studies, a genomic biomarker that predicts for high iBMV and development of brain metastases may be useful to inform recommendations for CNS surveillance and treatment in patients with NSCLC.

## Figures and Tables

**Table 1:** Patient characteristics.

	Entire Cohort	Positive iBMV	Neutral iBMV	Negative iBMV
*n*, %				
	312 (100%)	95 (30.4%)	197 (63.1%)	20 (6.4%)
Median age, years				
	67	67	68	66
Sex				
Male	155 (49.7%)	42 (27.1%)	102 (65.8%)	11 (7.1%)
Female	157 (50.3%)	53 (33.8%)	95 (60.5%)	9 (5.7%)
Race				
White	265 (84.9%)	77 (29.1%)	169 (63.8%)	19 (7.2%)
African American	40 (12.8%)	16 (40%)	24 (60%)	0
Asian Indian	1 (0.3%)	0	0	1 (100%)
Hispanic	1 (0.3%)	0	1 (100%)	0
Other	5 (1.6%)	2 (40%)	3 (60%)	0
Karnofsky Performance Status				
100–80%	201 (64.4%)	57 (28.3%)	132 (65.7%)	12 (59.7%)
70–50%	99 (31.7%)	35 (35.4%)	57 (57.6%)	7 (7.1%)
40–0%	9 (2.9%)	1 (11%)	7 (77.8%)	1 (11%)
Unknown	3 (1.0%)	2 (66.7%)	1 (33.3%)	0
Actionable Mutations				
EGFR	69 (22.1%)	21 (30.4%)	41 (59.4%)	7 (10.1%)
KRAS	94 (30.1%)	32 (34.0%)	56 (59.6%)	6 (6.4%)
ALK	19 (6.1%)	8 (42.1%)	7 (36.8%)	4 (21.1%)
ROS1	14 (4.5%)	6 (42.9%)	6 (42.9%)	2 (14.3%)
BRAF	27 (8.7%)	26 (96.3%)	1 (3.7%)	1 (3.7%)
Smoking Status				
Former	194 (62.2%)			
Current	69 (22.1%)			
Never	49 (15.7%)			
Stage				
I	4 (1.3%)			
II	3 (1.0%)			
III	38 (12.2%)			
IV	267 (85.6%)			
NSCLC Histology				
Adenocarcinoma	247 (79.2%)			
Squamous cell carcinoma	53 (17.0%)			
Large-cell carcinoma	2 (0.6%)			
NSCLC-NOS	10 (3.2%)			

## Data Availability

The data presented in this study are available on request from the corresponding author due to privacy and legal restrictions.
